# Maternal and infant microbiome and birth anthropometry

**DOI:** 10.1016/j.isci.2024.110312

**Published:** 2024-06-21

**Authors:** Swetha Padiyar, Vanishree Nandakumar, Swapna Kollikonda, Sreenivas Karnati, Naseer Sangwan, Hany Aly

**Affiliations:** 1 Neonatology, Wake Forest University School of Medicine, Winston-Salem, NC, USA; 2 Division of Neonatology, Cleveland Clinic Children’s, Cleveland, OH, USA; 3 Department of Obstetrics & Gynecology, Cleveland Clinic, Cleveland, OH, USA; 4 Shared Laboratory Resources (SLR), Lerner Research Institute, Cleveland Clinic, Cleveland, OH, USA; 5 Cardiovascular and Metabolic Sciences, Lerner Research Institute, Cleveland Clinic, Cleveland, OH, USA

**Keywords:** Medicine, Microbiome, Reproductive medicine

## Abstract

Preterm birth is the leading cause of neonatal mortality and morbidity. Microbiome dysbiosis in the mother and infant may contribute to their adverse outcomes. 16S rRNA amplicon sequencing was performed on all samples. Phyloseq, microbiomeSeq, and NetCoMi were utilized for bioinformatics analysis. Statistical tests included the Wilcoxon test, ANOVA, permutational multivariate analysis of variance (PERMANOVA), and linear regression. Statistical significance was set at *p* value <0.05. The establishment of an infant’s microbiome most likely begins *in utero* and is influenced by the maternal microbiome. Infants’ samples were enriched with *Salmonella*. There is a complex interplay among the microbial taxa noticeable at birth, exhibiting variability in interaction within the same host and across different hosts. Both maternal and infant microbiomes influence the anthropometric measures determined at birth, and a sex-based difference in correlation exists. This study highlights the potential role of maternal and infant microbiomes in improving pregnancy and neonatal outcomes.

## Highlights

• The process of establishment of the infant microbiota may begin *in-utero*
• Preterm infants born between 28 and 32 weeks exhibit an abundance of *Salmonella*
• The maternal and infant microbiome influence anthropometry measured at birth
• Polymicrobial interactions are noticeable at birth and exhibit variability

## Graphical abstract

**Figure:**
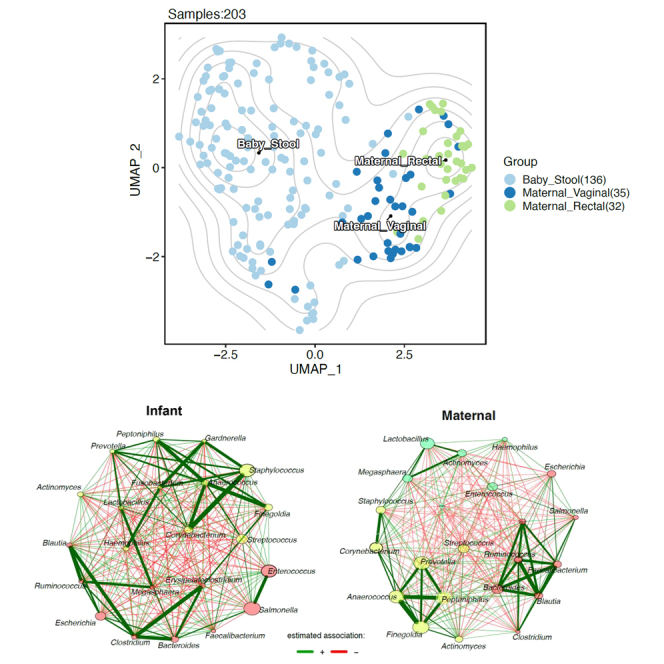

## Introduction

Globally, each year, over 13 million infants are born prematurely,^1^ with about 10% of them delivered between 28 and 32 weeks.^2^ Preterm birth (<37 weeks of gestation) accounts for one million deaths yearly and is still the primary cause of neonatal mortality.^1^ The surviving infants are predisposed to short and long-term complications enduring into adulthood, posing a significant socioeconomic and emotional burden.^3^^,^^4^ Although the exact reason for preterm birth is unclear, maternal factors like smoking, poor nutrition, obesity, cardiometabolic diseases, infections, pre-eclampsia, and medication use, including antibiotics, are identified as risk factors.^5^ Over one-third of premature births are attributed to intrauterine infections originating from the microbiota of the gut and urogenital tract.^5^ The alteration in the normal equilibrium of the microbiome (dysbiosis) in gestational morbidities has been speculated as a likely cause for preterm labor, but the evidence remains inconclusive.^5^^,^^6^

The term microbiome refers to the collective genome of the microbiota (communities of bacteria, viruses, fungi, and archaea) that are in symbiotic relationships within the human body and have an important role in the homeostasis of normal physiologic, metabolic, and immune functions contributing to the overall health and well-being.^7^^,^^8^ Early life microbiomes play a significant role in digestion, growth, and neurobiological development, setting the stage for adult microbiome and long-term health and disease.^9^^,^^10^^,^^11^^,^^12^ The process of microbiome colonization is continuous and dynamically changing in infancy and early childhood.^13^ Compared to full-term infants, premature infants have significantly different microbiomes with a decreased abundance of beneficial microbes.^14^ Microbiome dysbiosis in preterm infants has been linked not only to short-term adversities like sepsis and necrotizing enterocolitis but also to long-term consequences like asthma, obesity, autism, and immunological adversities.^10^

The neonatal period (birth-28 days) is identified as a key phase for the neurophysiological growth and maturation of the infant and the colonization and organization of a healthy microbiota.^9^^,^^15^ The physical and neurological growth and maturation of the fetus continue into the third trimester. As a result, infants born prematurely will be stunted, with the potential for an imbalance of the microbiome equilibrium. In addition to the low birth weight, preterm infants have different body composition and nutritional requirements compared to their term-born peers.^16^ Anthropometric measurements such as birth weight (BW), birth length (BL), and birth head circumference (HC) are often used in neonatal intensive care units (NICUs) to identify nutritional requirements and assess growth and developmental risks in infants. BW is an important predictor of neonatal mortality and outcomes and HC is an indicator of neuronal maturation and brain development.^17^^,^^18^ Although microbiome dysbiosis during pregnancy has been implicated in exerting an indirect effect on the gestational age and birth weight,^19^ it remains uncertain if the maternal microbiome directly influences the birth anthropometry of the infants.

It is still unknown if preterm infants inherit dysbiotic microbiome from the mother or if preterm birth causes disruption in the establishment of the normal microbiome. There is an ongoing debate about the timing of the microbiome seeding in a neonate, with differing opinions if the establishment occurs prenatally or at birth. The diverse perspectives with the existing evidence supporting both viewpoints contribute to the complexity of understanding the precise onset of the microbiome. The influence of the maternal microbiome on fetal growth and development during pregnancy and on the infant’s microbiome in the postnatal period has been poorly understood. The maternal microbiome profile in the context of preterm birth has also been understudied. The association of maternal and infant microbiome and microbial interactions has not been demonstrated. With this study, we aim to address these knowledge gaps.

This prospective cohort study aims to understand the influence of maternal microbiome on infant microbiome and correlate it with the BW, HC, and BL in preterm infants.

### Methods

This prospective study enrolled all infants born between 28 and 32 weeks of gestation and their mothers. Infants <28 and >32 weeks of gestation and those with known genetic and congenital defects were excluded from the study. Informed consent was obtained for all participants. The study was approved by the Cleveland Clinic Institutional Review Board and was conducted from 1^st^ June 2021 through 31^st^ August 2022.

#### Clinical data collection

Electronic medical records were used to collect maternal data, including, but not limited to gravida, parity, body mass index (BMI), diabetes, hypertension, preeclampsia, group B Streptococcus (GBS) status, preterm membrane rupture, infections, and antibiotic use. Neonatal data collected were baseline demographic data, birth history, sex, weight, length, and head circumference at birth. *Z* scores were collected from Fenton’s growth charts for anthropometric measurements at birth on admission to the NICU.^20^

#### Microbiome sample collection

Maternal vaginal and rectal swabs were collected at the time of delivery, either immediately prior to delivery or within 24 h of delivery. Infant’s first fecal samples were collected from the diaper using a sterile Q-tip at birth and placed in a sterile medium container. Samples were immediately frozen at ˗80°C to be used for molecular analysis. Total bacterial community was characterized using 16S rRNA amplicon sequencing methods published by our group previously.^21^^,^^22^^,^^23^^,^^24^^,^^25^^,^^26^^,^^27^^,^^28^^,^^29^

#### Bioinformatics and statistical analysis

Sequencing data from the 16S rRNA gene amplification was analyzed using Divisive Amplicon Denoising Algorithm (DADA2) pipeline.^30^ Analysis of the microbiome at birth adjusting for gestational age in linear regression models in R, with bacteria quantity and other continuous variables, were transformed to meet model assumptions. Batch correction was achieved with ComBat.^31^ White’s non-parametric t test and ANOVA post hoc analysis were performed. A semi-parametric rank-based approach for inference in a graphic model (SPRING) from NetCoMi (network construction and comparison for microbiome data) package in R^32^ was used to perform network analysis. Node sizes were scaled using Eigenvector centrality.

Differential abundance test benchmarking was performed using DAtest package (https://github.com/Russel88/DAtest/wiki/usage#typical-workflow). Briefly, differentially abundant methods were compared with false discovery rate (FDR), area under the (receiver operator) curve (AUC), empirical power (power), and false positive rate (FPR). Based on the DAtest’s benchmarking, we selected lefseq and ANOVA as the methods of choice to perform differential abundance analysis. We assessed the statistical significance (*p* < 0.05) throughout. Whenever necessary, we adjusted *p* values for multiple comparisons according to the Benjamini and Hochberg method to control FDR (Benjamini and Hochberg, 1995).

SAS/STAT version 9.4 PROC POWER was used to perform power calculations. A separate mixed measures model was used to assess associations between weight, height, and length *Z* scores. All tests were two-tailed for a significance of 0.05.

## Results

### Patient characteristics

The study included 40 mother-infant dyads, of which 36 were singleton pregnancies and 4 were twin gestation. Eleven mothers were primigravida, and 27 were primipara. Six mothers (6/36) had a history of substance abuse, of which 5 mothers consumed marijuana. Approximately 64% (*n* = 23) of mothers had pre-existing health conditions, of which conditions related to mental health (generalized anxiety and depression) were most common (7/23), followed by cardiorespiratory conditions (4/23) such as sinus arrhythmia, asthma, pulmonary hypertension, and heart failure. None of the mothers had any history of smoking or alcohol use during pregnancy. All mothers received antenatal steroids and prophylactic antibiotics.

Of the 40 infants in the study, 25 were born via cesarian section (62.5%). The study population included more female infants (*n* = 23; 57.5%). Maternal and infant characteristics are summarized in Table 1.

**Table 1:** Study population characteristics

Maternal characteristics (*n* = 36)	
Age (years)∗	29 ± 6
Race (%)	
White	17 (47.2%)
Black	14 (38.8%)
Asian	1 (2.7%)
Hypertension (%)	
Gestational	10 (27.7%)
Chronic	3 (8.3%)
Preeclampsia	14 (38.8%)
Gestational diabetes	5 (13.8%)
Chorioamnionitis	16 (44.4%)
PPROM	21 (58.3%)
Antenatal steroids	36 (100%)
Placental abruption	7 (19.4%)
Placenta previa	2 (5.5%)
Maternal BMI∗ (kg/m^2^)	34.72 ± 9.2
**Mode of delivery**	
Vaginal	15 (37.5%)
**Infant characteristics (*n* = 40)**	
Sex, male	17 (42.5%)
Gestational age∗	30 ± 1.3
Birth anthropometry∗	
Weight (g)	1475 ± 380
Length (cm)	39.7 ± 3
Head circumference (cm)	28 ± 2
Birth anthropometry∗ (*Z* scores)	
Weight	0.01 ± 0.87
Length	0.01 ± 0.87
Head circumference	−0.06 ± 0.99

Data are expressed in number (%) except with ∗ data are expressed in mean ± standard deviation (SD). BMI: body mass index, PPROM: preterm premature rupture of membranes.

### Maternal and infant microbial diversity and taxonomic composition

**Figure 1:**
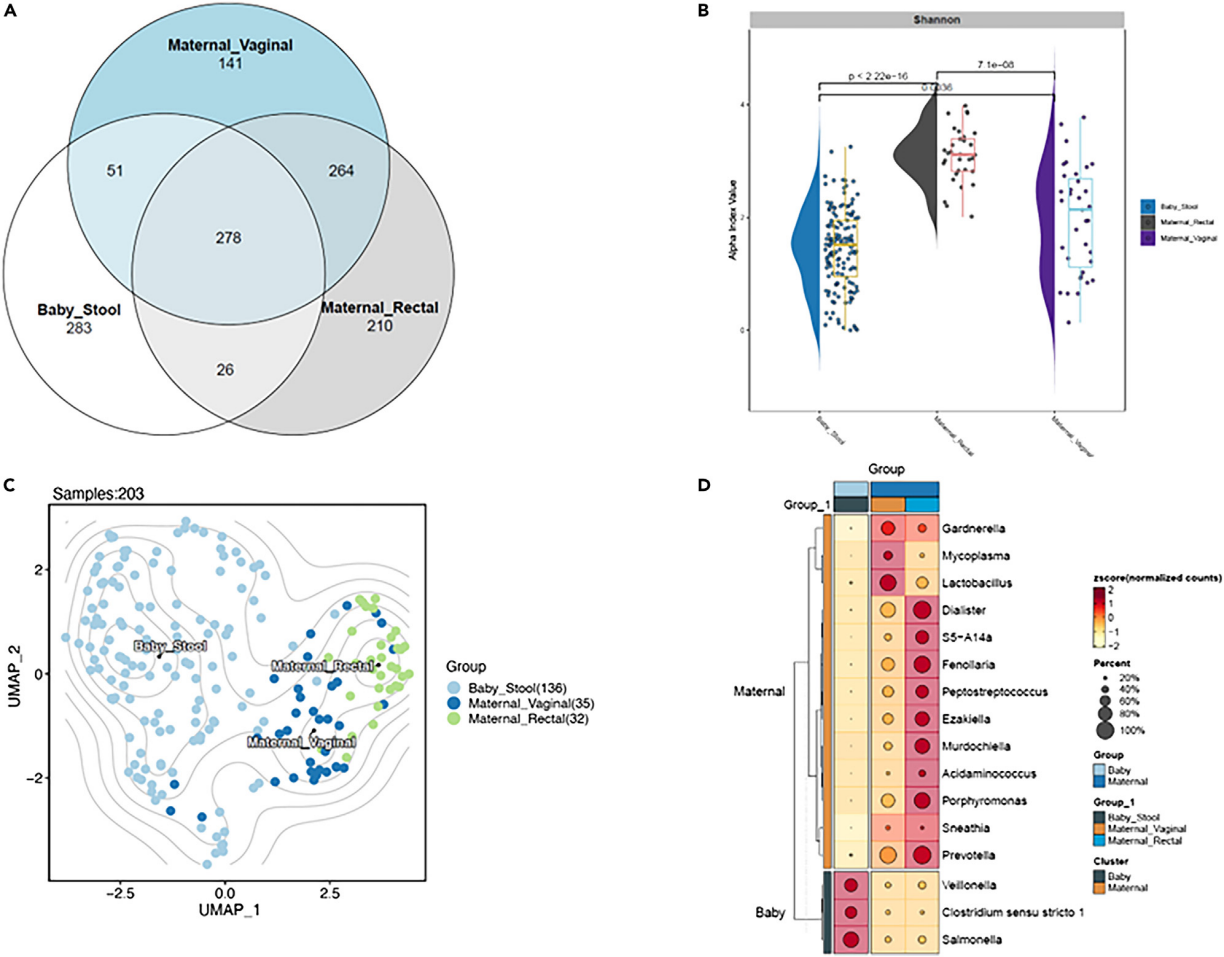
Maternal and infant microbial diversity and taxonomic composition (A) Venn diagram demonstrating the distribution of amplified sequence variants (ASVs) between maternal and infant samples. (B) Bacterial α diversity measured by the Shannon index. Intergroup *p* values are calculated by Wilcoxon signed rank test. (C) Uniform manifold approximation and projection (UMAP) demonstrating dimension reduction analysis of β diversity distance matrices (distance = Bray Curti’s distances, PERMANOVA, *p* < 0.01). (D) Differential abundance heatmap for microbial markers in maternal and infant samples demonstrating hierarchical clustering and normalized abundance (*Z* scores).

The DADA 2 pipeline identified a total of 1,253 amplified sequence variants (ASVs). Of these, 283 ASVs (44% of infant’s ASVs and 22% of all ASVs) were unique to the infant, and 141 ASVs (19% of maternal vaginal and 11% of all ASVs) were unique to the mother’s vaginal samples. A total of 278 taxa were common to all three sources. Upon downstream analysis of the ASV abundance matrix, a significant overlap was observed between maternal and infant microbiomes, with a higher degree of overlap of the maternal vaginal ASVs compared to the rectal ASVs (vaginal = 51 vs. rectal = 26; Figure 1A).

Alpha (α) diversity measured by the Shannon index assesses microbial diversity, including the number of taxa (richness) and the relative abundance (evenness). The α diversity was highest in maternal rectal samples, followed by maternal vaginal samples. Infant samples demonstrated a lower α diversity compared to the maternal samples (Figure 1B). The Wilcoxon signed rank test determined the statistical differences between α diversity patterns. The α diversity between infant stool and maternal samples (vaginal [*p* = 0.0036], rectal [*p* < 0.0001]) and among the maternal (rectal and vaginal, *p* < 0.0001) were statistically significant (Figure 1B). Dimension reduction analysis of the beta (β) diversity (identity of the taxa) distance matrices (distance = Bray Curti’s distances, PERMANOVA, *p* value <0.01) of the microbiome data with uniform manifold approximation and projection (UMAP) was performed. Three distinct clusters were noted for maternal vaginal, rectal, and infant samples. We noted that the maternal vaginal and rectal samples had similar microbial profiles. Compared to the rectal microbiome, maternal vaginal microbiome shared a greater degree of similarity with the infant’s microbiome (Figure 1C). Further, three main bacterial phyla, Bacillota (Firmicutes), Bacteriodota (Bacteroidetes), and Pseudomonadota (Proteobacteria) by 16S rRNA gene amplicon sequencing analysis were identified across all samples. Hierarchical clustering and normalized abundance (*Z* scores) of genus-level maternal and infant microbiomes are indicated in Figure 1D. Maternal rectal samples showed a higher relative abundance of *Prevotella*, *Dialister*, and *Porphyromonas* compared to the vaginal samples. Maternal vaginal samples were abundant for *Lactobacillus* and *Gardnerella*. *Salmonella* was predominant in infants’ stool samples (Figure 1D).

### Network analysis identifying the correlation between maternal and infant microbiome

**Figure 2:**
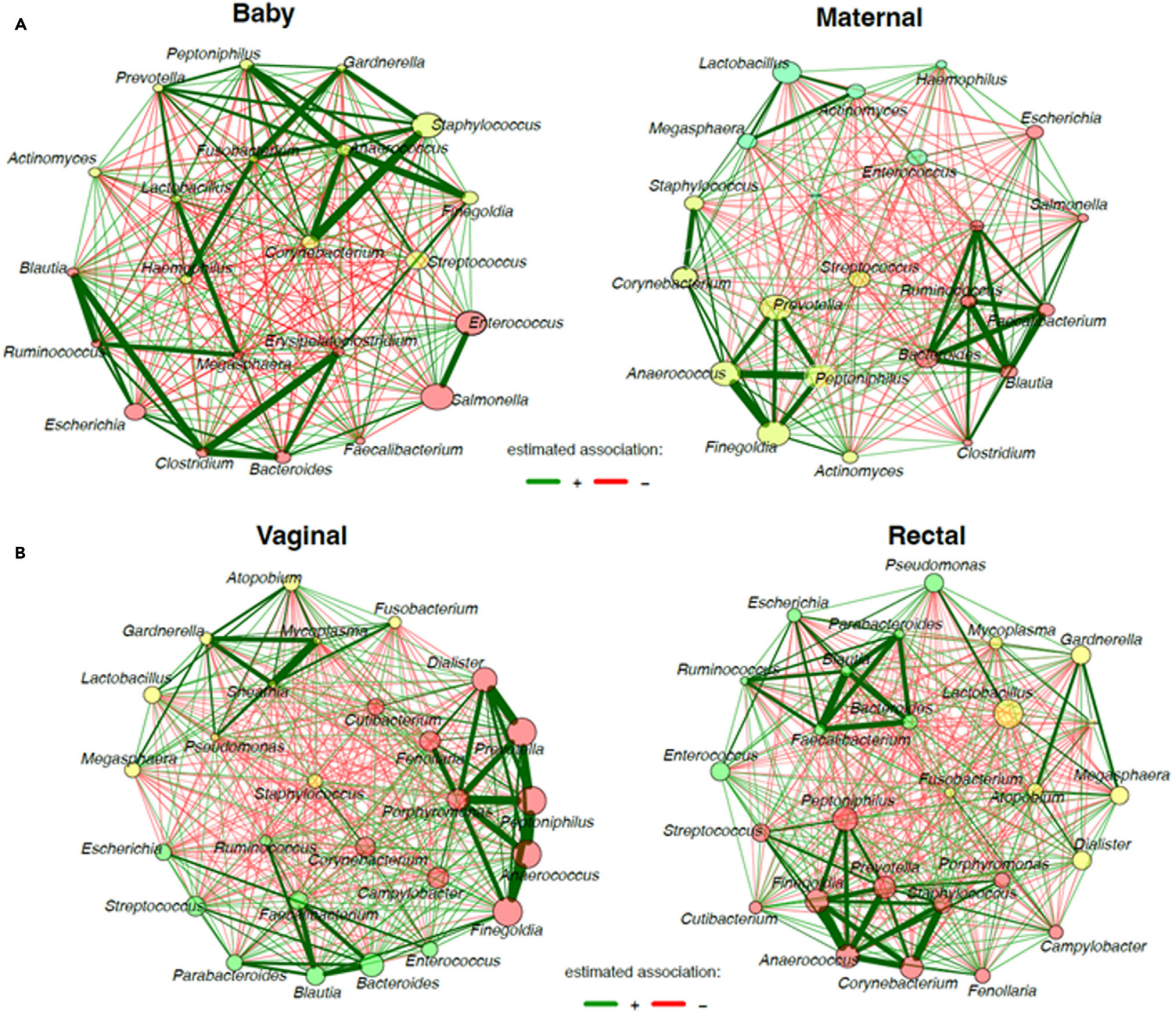
Differential network analysis of the mother and infant microbiome Node sizes are proportional to the taxon’s sum of the modified centered log ratio (mclr). Clusters with two more shared taxa have the same color in both networks. Signed and weighted relationships are shown. Green edges correspond to positive associations, and red edges represent negative associations. Node size denotes relative abundance. (A) Network comparison between the infant and maternal microbial genera. (B) Network comparison between the maternal rectal and vaginal microbial genera.

Differential association networks were constructed to study the microbial interactions in maternal and infant samples. In the infant’s sample, a strong positive correlation was noted between *Staphylococcus*, *Gardnerella*, *Corynebacterium*, *Prevotella*, *Lactobacillus*, *Megasphaera*, *Escherichia*, *Clostridium*, and *Bacteroides*. Weak negative correlations were noted between *Lactobacillus*, *Enterococcus*, *Salmonella*, and *Escherichia*. *Lactobacillus* was positively associated with *Actinomyces*, *Megasphaera*, and *Staphylococcus in* the maternal microbiome. The maternal sample noted weak negative correlations with *Streptococcus*, *Staphylococcus*, *Lactobacillus*, *Enterococcus*, bacteria, and *Salmonella.* Further, in the maternal vaginal sample, weak negative correlations were noted with *Staphylococcus*, *Lactobacillus*, *Prevotella*, *Escherichia*, and *Pseudomonas* while strong positive correlations was noted with *Prevotella*, *Dialister*, *Anaerococcus*, *Finegoldia*, *Gardenrella*, and *Mycoplasma*. In the maternal rectal samples, strong positive associations were noted with *Staphylococcus*, *Prevotella*, *Anaerococcus, Finegoldia*, *Bacteroides*, *Blautia*, and *Faecalibacterium*. We observed a difference in correlations of the microbial genera between the maternal and infant samples and among the maternal rectal and vaginal samples (Figure 2).

### Maternal and infant microbial differences stratified by gestational pathologies

Differential abundance analysis (metastat, *p* < 0.001) for taxonomic features across maternal gestational morbidities demonstrated that mothers with diabetes showed a higher abundance of *Staphylococcus*, *Faecalibacterium*, and *Clostridioides*, compared to non-diabetic mothers (Figure S1B). Mothers with preterm premature rupture of membranes (PPROM) and infection showed a higher abundance of *Streptococcus*, while mothers with chorioamnionitis showed a higher abundance of *Ralstonia* (Figures S1A and S1B). *Lactobacillus* was abundant in mothers with chronic hypertension, whereas those with gestational hypertension showed abundance in *Parvimonas* and *Azotobacter* (Figure S1B). Relatively healthy mothers without gestational morbidities had higher *Bifidobacterium and Lactobacillus* counts (Figure S1).

**Figure 3:**
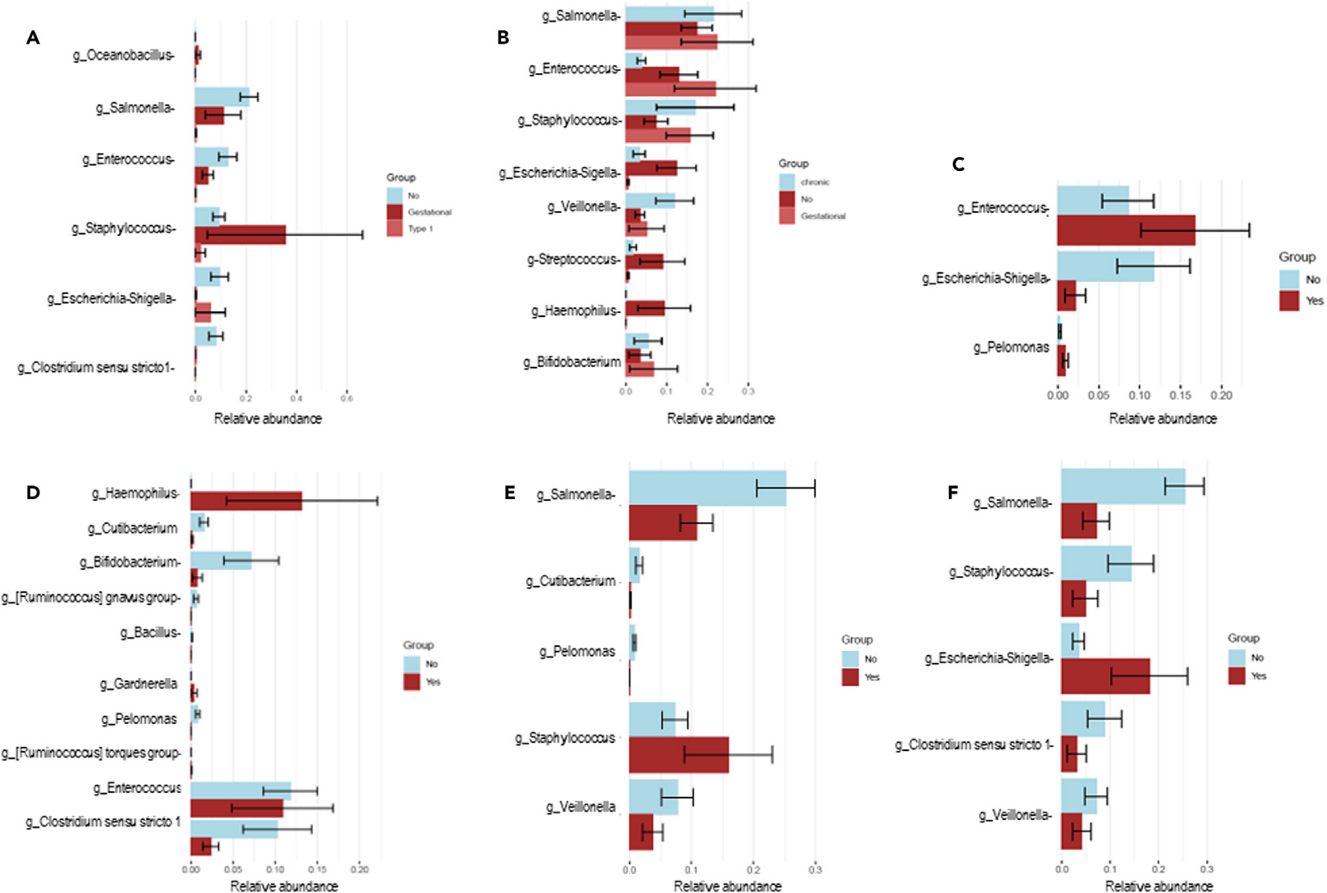
Infant microbial differences in gestational morbidities (A–F) Microbial relative abundancies are depicted in each of the morbidities, (A) diabetes; (B) hypertension; (C) preecclampsia; (D) chorioamnionitis; (E) preterm premature rupture of membranes (PPROM); (F) infections. Relative abundancies in infants born to mothers with (red) and without (blue) gestational morbidities are shown.

Infants born to mothers with chorioamnionitis showed abundance of *Haemophilus* and *Enterococcus*. Infants of diabetic mothers showed higher *Staphylococcus* compared to infants of mothers with hypertension and infection (Figure 3). Infants with a maternal history of hypertension showed an abundance of *Salmonella*, *Enterococcus*, and *Staphylococcus*. *Escherichia-Shigella* was significantly higher in abundance in infants with maternal infection, while *Staphylococcus* was higher in infants with a history of PPROM. *Salmonella* was significantly enriched in infants born to relatively healthy mothers (Figure 3). Based on the mode of delivery, infants born via C-section had higher counts of *Staphylococcus* and *Escherichia-Shigella*, whereas those born vaginally had an abundance of *Streptococcus* and *Haemophilus* (Figure S2).

**Figure 4:**
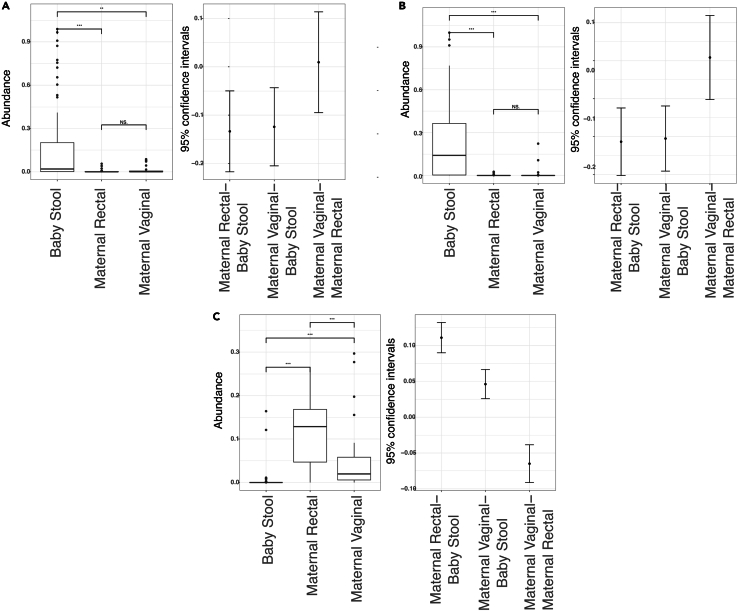
Post-hoc analysis across maternal and infant microbiome samples (A) *Staphylococcus*. (B) *Salmonella*. (C) *Prevotella*. Confidence intervals are plotted next to the abundance boxplots.∗∗*p* < 0.01; ∗∗∗*p* < 0.001; NS: non-significant. False discovery rate (FDR) correction has been achieved by Benjamini-Hochberg method.

Post hoc analysis performed at 95% confidence intervals (CI) showed significant results between infant stool samples and maternal vaginal samples for *Staphylococcus* (*p* < 0.01), *Salmonella* (*p* < 0.001), and *Prevotella* (*p* < 0.001). Results were also significant between the infant stool and maternal rectal samples (*p* < 0.001). Between the maternal rectal and vaginal samples, significance was noted with *Prevotella* only (*p* < 0.001) (Figure 4).

### Microbiome and anthropometry measured at birth

**Figure 5:**
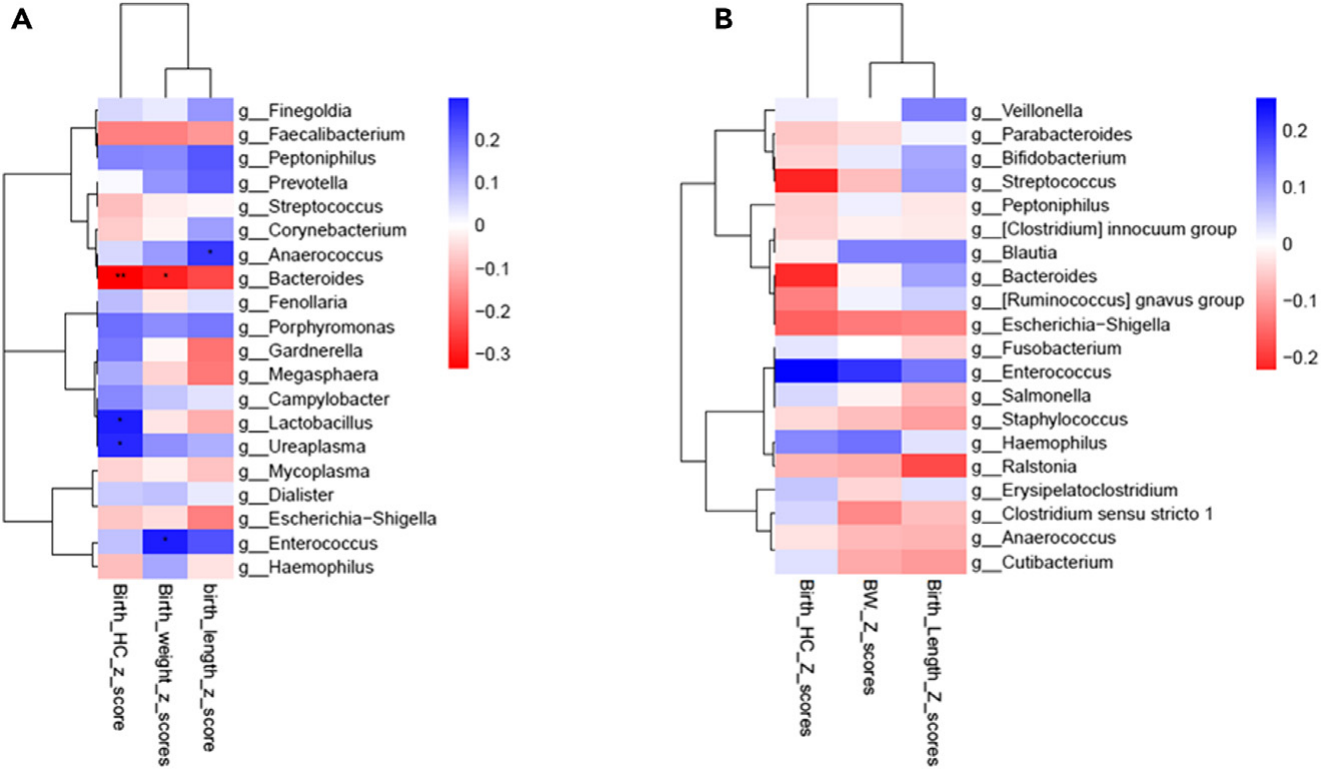
Correlation of microbiota with infant z scores for birth head circumference (HC), birth weight (BW), and birth length (BL) (A) Correlation of maternal microbiota with z scores. (B) Correlation of infant microbiota with z scores. Red indicates negative correlation and blue indicates positive correlation; ∗*p* < 0.05, ∗∗*p* < 0.01, ∗∗∗*p* < 0.001. False discovery rate (FDR) correction has been achieved by Benjamini-Hochberg method.

Next, we investigated the influence of maternal and infant microbiomes on the infant’s z scores for BW, BL, and HC. Correlation of maternal microbiome genera with anthropometry measured at birth demonstrated a positive correlation between *Lactobacillus* and *Ureaplasma* and HC, *Enterococcus* and BW, *Anaerococcus* and BL. *Bacteroides* correlated negatively with *Z* scores for all birth anthropometric measurements (Figure 5A). Among the infants, a positive correlation was noted with the genus *Enterococcus*, and a negative correlation was noted with *Escherichia/Shigella* and *Staphylococcus*. While *Streptococcus and Bacteroides* negatively correlated with HC measured at birth, a positive correlation was noted with BL measured at birth (Figure 5B).

**Figure 6:**
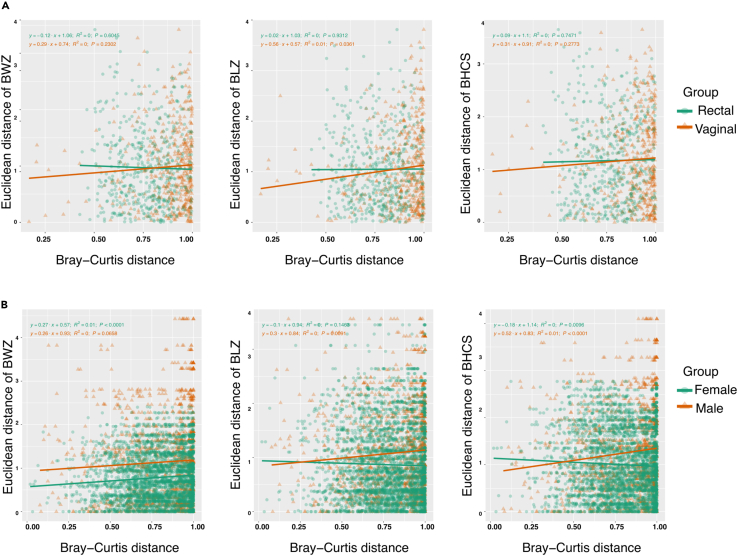
Comparison of infant growth matrices with beta-diversity distance matrices (A) Maternal rectal and vaginal microbiome and birth anthropometry. (B) Sex-based correlation of birth anthropometry and infant’s microbiome BWZ: *Z* scores for birth weight; BLZ: *Z* scores for birth length; BHCS: *Z* scores for birth head circumference.

To better understand the relationship between maternal and infant microbiome and birth anthropometry, distance matrices were compared with permutational multivariate analysis of variance (PERMANOVA). Continuous variables (BL, BW, and HC) were assessed with Euclidian distance, and microbiome dissimilarity was calculated with Bray-Curti’s distance for maternal and infant microbiomes. Maternal vaginal microbiome and birth anthropometry showed a positive correlation with all anthropometric measures. However, significance was noted only with BL (*p* = 0.036) (Figure 6A). Further, the correlation between sex-based differences in the microbiome and birth anthropometry showed that the microbiome of male infants correlated positively with BL (*p* = 0.0091) and HC (*p* < 0.0001). The microbiome of the female infants correlated positively with BW (*p* < 0.0001) and negatively with HC (*p* = 0.0096) (Figure 6B).

## Discussion

We extensively studied the microbiome in the preterm infant-maternal cohort with gestational ages between 28 and 32 weeks. We observed that the preterm infant’s microbiome is significantly diverse at birth and overlaps with the maternal rectal and vaginal microbiome. We identified 22% of the ASVs that were unique to the infant population, which points to the *in-utero* initiation of microbiome acquisition. This is the first study to demonstrate the complex polymicrobial interactions between the taxa in preterm infants-maternal cohort. Our analysis illustrates that the complex polymicrobial interactions are noticeable at birth. We also noted that the interactions among the microbial taxa exhibit variability within the same host and across different hosts. We also demonstrated that an infant’s anthropometry, measured at birth, is influenced by the maternal and infant’s microbiome.

Bacillota, Bacteriodata, Psudomonadota, and Actinomycetota were the main phyla observed in the maternal population. Consistent with previous evidence, we found a lower vaginal α diversity compared to the rectal samples, along with an overlap of maternal rectal and vaginal microbiome.^33^^,^^34^ In our study, although maternal vaginal microbiome was enriched with *Lactobacillus* and *Gardnerella*, all mothers delivered prior to 32 weeks of gestation. Contrary to prior evidence, we did not observe that the cooccurrence of *Lactobacillus* and *Prevotella* affected the duration of gestation.^35^ However, our study strengthened the previous evidence that *Prevotella*, *Staphylococcus*, *Streptococcus*, and *Mycoplasma* in the vaginal microbiome are associated with an increased risk of preterm delivery.^5^^,^^35^^,^^36^ Further, we observed that the mothers with gestational morbidities showed an abundance of *Staphylococcus*, *Faecalibacterium*, *Streptococcus*, and *Ralstonia*, while the relatively healthy mothers in the study population had higher probiotic bacteria like *Bifidobacterium and Lactobacillus*^37^^,^^38^ emphasizing that microbial dysbiosis occurs in gestational pathologies.^39^^,^^40^ We observed a higher abundance of *Streptococcus* in the mothers with infection and PPROM. *Streptococcus* is the leading cause of adverse maternal outcomes and neonatal infections. Asymptomatic colonization is seen in 1 in 4 pregnant women^41^ and all women are screened between 36 and 38 weeks^42^ Although probiotics administration during pregnancy has reduced the colony counts of *Streptococcus*, the benefits noted were closer to term (35–37 weeks).^43^ Additionally, we found a positive correlation between *Streptococcus* and *Lactobacillus* in our maternal cohort, undermining the benefits of *Lactobacillus*-containing probiotics in mothers delivering preterm. However, the competitive correlations between *Lactobacillus* and *Enterococcus*, *Prevotella*, *Salmonella*, and *Escherichia* in the maternal population suggest that probiotics may still be beneficial. Though inconclusive benefits are noted with probiotic supplementation during pregnancy on the length of gestation and infant’s birth weight,^40^ it remains an area of ongoing research.^44^^,^^45^

In the neonatal population, consistent with the previous evidence,^46^^,^^47^ we observed an abundance of facultative anaerobes—*Salmonella*, *Enterococcus*, and *Staphylococcus*. Additionally, we observed that the infants born via C-section acquire common skin commensals^48^ and demonstrate a lower abundance of vaginal microbiota. However, contrary to previous evidence,^49^ we noticed that the abundance of *Haemophilus* was higher in vaginally born infants compared to infants born via C-section. Evidence suggests that term infants born via C-section are at risk of immune disorders, allergies, and metabolic disorders due to the difference in the acquired microbiota and a lower diversity of gut microbiota at one month of age.^50^^,^^51^ The risk of adverse events is higher in infants born preterm. Evidence suggests that vaginal microbial transfer may benefit C-section born-term infants.^50^^,^^52^ However, this may have adverse consequences in preterm infants, given the immaturity of the immune system and increased risk of adversities. Furthermore, we noted a difference in the microbial correlations between the mother and infants among the same genera. This indicates that the microbes interact differently in different hosts, indicating that the microbial transfer may not benefit preterm infants.

In addition to an immature immune system, preterm infants are at risk for growth failure and neurodevelopmental abnormalities. Birth anthropometry is not only a predictor of neonatal outcomes but also helps identify infants at risk for abnormal psychosomatic development. We observed a negative correlation between all anthropometric measurements and *Bacteroides* in the maternal microbiome. *Bacteroides* play a key role in maternal metabolic and immune function homeostasis and fetal growth.^53^^,^^54^ The genus has also been associated with excessive weight gain in pregnancy and preterm birth, and an increased abundance is noted during the third trimester.^19^^,^^55^^,^^56^ We hypothesize that *Bacteroides* may have a role in contributing to insulin resistance in pregnancy, and this, in turn, may influence the infant’s birth anthropometry. Recent evidence suggests that the maternal microbiome during the second trimester influences the birth weight of the infant by affecting glucose metabolism.^57^ Intestinal immunoglobulin A (IgA) regulates the inflammatory pathway-mediated insulin resistance, and animal models have demonstrated lower IgA cells in obesity.^57^ Additionally, insulin resistance increases in pregnancy, especially in the third trimester, even among healthy women, increasing the risk of adverse pregnancy and fetal and neonatal outcomes with long-lasting effects.^58^^,^^59^ Exercise and diet have been shown to improve outcomes in obese women and reduce insulin requirements in women with gestational diabetes.^58^^,^^60^ Evidence suggests that exercise influences gut microbiota. Preclinical studies have found that hormones that mediate appetite and hunger, like serum leptin and ghrelin, correlated negatively with *Bacteroides* and positively with *Lactobacillus*.^61^ In addition, the levels of short-chain fatty acids (SCFA) released by the gut microbiota, like *Bacteroides*, are increased in metabolic syndrome associated with low physical activity and poor nutrition and have been implicated in complications associated with insulin resistance in pregnancy.^62^^,^^63^

Further, in correlation of the infant’s anthropometry, measured at birth, and the infant’s microbiome, we observed that *Escherichia-Shigella* and *Staphylococcus* correlated negatively with the infant’s birth anthropometry. In preterm infants, the *Escherichia* genus is associated with early-onset sepsis; *Staphylococcus* has been associated with late*-*onset sepsis.^64^ Neonatal infections have been associated with a higher risk of bronchopulmonary dysplasia and cerebral palsy.^65^ Both strains, although early colonizers, are associated with prolonged hospitalization, adverse growth, and neurodevelopment in preterm infants.^47^ Animal experiments suggest that the abundance of *Staphylococcus* in preterm gut and antibiotic exposure disrupts the mucosal barrier and dysregulates the immune responses, increasing the susceptibility to infections.^64^ Gut microbiome has been linked with normal growth and maturation of the gastrointestinal tract (GIT).^66^ Further, there is an interplay between the developing GIT and the brain via the gut-brain axis.^67^ Aligning with this, we observed that *Streptococcus*, *Escherichia-Shigella*, and *Bacteroides* in the infant’s microbiome correlated negatively with HC. Most of the fetus’s weight gain and cerebral maturation occurs in the third trimester, and preterm infants have an attenuation of the gut’s normal digestive and absorptive functions, which may impact the developing brain, leading to low BW and HC. We hypothesize that the maternal microbiota and the fetal gut microbiota established *in utero* influence fetal weight gain and psychosomatic growth, thus influencing birth anthropometry. Further, we also noted sex-based differences in correlation between the infant’s microbiota and the birth anthropometry. This may be in support of the preclinical evidence suggesting sex-based differences in the molecular mechanisms underlying the metabolic pathways.^59^^,^^68^ Further research is required to determine the exact timing of the onset of fetal microbiota and the development of the gut-brain axis and its influence on the metabolic pathways. These findings support the hypothesis that maternal and infant microbiota influences birth anthropometry. Healthy lifestyle habits, including a well-balanced diet and regular physical exercise, must be emphasized early in antenatal care to maintain microbial homeostasis and improve maternal and neonatal outcomes.

Our study has several merits. Our study is the first to demonstrate polymicrobial interactions at birth and perform differential network analysis. We also explored the relationship between maternal and infant microbiomes on BW, BL, and HC and stratified the maternal and infant microbiomes in gestational morbidities. We found ASVs that were unique to the infant, suggesting the *in-utero* establishment of the microbiome, and further demonstrated the sex-based differences in the influence of the infant’s microbiome, suggesting an effect on the metabolic pathways.

### Limitations of the study

Our prospective study included only preterm infants. However, this study was based on the previous knowledge of the microbiome being different in term-born and preterm infants.^14^^,^^46^ Although our study sample was small, it was adequately powered. Maternal samples were obtained only at the time of delivery. Longitudinal studies with sample collection at regular intervals throughout the pregnancy are needed to outline the temporal microbiome changes. Our study included maternal rectal and vaginal samples only. Future studies must plan to incorporate amniotic fluid, placental samples, and breastmilk samples to obtain a complete overview of the source of the infant’s microbiome. The study inherited a limitation related to the timing of the first stool in preterm infants; some infants may not stool on the first day of life. Therefore, there is no definitive method to verify if the infant’s gut was colonized *in utero*, after birth, or both. In such cases, the influence of the environmental factors cannot be excluded. Furthermore, commonly seen NICU environmental colonizers are *Staphylococcus*, *Streptococcus*, and *Niesseria*.^69^ On the contrary, *Salmonella* was the predominant microbe in our study population. The overlap between the maternal and infant’s microbiome and the significant α diversity of the ASVs unique to the infant suggest the possibility of *in-utero* colonization of the infant’s gut. We studied the microbiome’s influence on birth anthropometry only. As the neonatal microbiome undergoes dynamic changes after birth, further research must explore the maternal and infant microbiome on growth, neonatal morbidities, and neurodevelopment.

### Conclusion

This study suggests the establishment of the gut microbiome in neonates may begin *in utero* and is further influenced by the mode of delivery. The influence of maternal and infant microbiome on the infant’s birth weight, length, and head circumference underscores that the *in-utero* microbiome is important for improved pregnancy and neonatal outcomes. Gestational morbidities possibly will cause a shift in the normal microbiome homeostasis. Preterm infants likely inherit a dysbiotic microbiome and thus are at an increased risk of adverse neonatal outcomes. Further studies that longitudinally follow microbiome changes in preterm infants may provide better insight into the role of the microbiome in growth and neurodevelopment during infancy.

## Resource availability

### Lead contact

Further information and requests should be directed to and will be fulfilled by the lead contact, Hany Aly, MD (alyh@ccf.org).

### Materials availability

This study did not generate new unique reagents.

### Data and code availability

• All data reported in this paper will be shared by the lead contact upon request
• This paper does not report original code
• Any additional information required to reanalyze the data reported in this paper is available from the lead contact upon request.

## Acknowledgments

Mark Lauer Pediatric Research Grant.

## Author contributions

S.P.: funding acquisition, data curation, writing- original draft, review, and editing. V.N.: methodology, data curation and visualization, writing- original draft, review, and editing. S. Kollikonda: consent collection, data curation, methodology, writing-review and editing. S. Karnati: funding acquisition, data curation, methodology, writing-review and editing. N.S.: data curation, formal analysis, investigation, software, validation, visualization, and writing– review and editing. H.A.: conceptualization, funding acquisition, supervision, validation, visualization, writing-review and editing.

## Declaration of interests

The authors declare no competing interests

## STAR★Methods

### Key resources table

**Table:** 

REAGENT or RESOURCE	SOURCE	IDENTIFIER
**Biological samples**		

Maternal vaginal and rectal samples	Study patients	
Infant stool samples	Study patients	

**Deposited data**		

Data reported in this paper is available from the lead contact upon request.		

**Software and algorithms**		

Divisive Amplicon Denoising Algorithm (DADA2) pipeline		https://benjjneb.github.io/dada2/tutorial_1_8.html
Semi-Parametric Rank-based approach for Inference in a Graphic model (SPRING)	NetCoMi	Peschel et al.^32^
Differential abundance analysis	DAtest package	https://github.com/Russel88/DAtest/wiki/usage#typical-workflow
Microbiome data analysis	phyloseq	https://joey711.github.io/phyloseq/

### Experimental model and study participant details

Participant details can be found in methods, results, and Table 1.

This study enrolled all infants born between 28 and 32 weeks of gestation and their mothers. Infants <28 and >32 weeks of gestation and those with known genetic and congenital defects were excluded from the study. Informed consent was obtained for all participants. The study was approved by the Cleveland Clinic Institutional Review Board and was conducted from 1^st^ June 2021 through 31^st^ August 2022. The mothers in the study belonged to White, Black, and Asian populations with an average age of 29 ± 6. Newborn infants were identified anatomically as belonging to male and female sex.

### Method details

#### 16S rRNA gene sequencing and bioinformatics analysis

Maternal vaginal and rectal swabs were collected at the time of delivery, either immediately prior to delivery or within 24 h of delivery. Infant’s first fecal samples were collected from the diaper using a sterile Q-tip at birth and placed in a sterile medium container. Maternal and infant samples were labeled, and the samples were immediately frozen at −80^°^C to be used for molecular analysis.

DNA isolation from the biospecimens was performed using Qiagen PowerFecal Pro kits. A nested polymerase chain reaction (PCR) amplification for the V3-4 regions of the 16s rRNA gene was performed using Illumina MiSeq platform. Illumina iSeq 100 with a 2x 150 read length was used to perform amplicon sequencing. 16S rRNA gene amplicon sequencing and bioinformatics analysis were performed using methods explained earlier. Briefly, raw 16S amplicon sequence and metadata, were *demultiplexed using split_libraries_fastq.*py script implemented *in QIIME2*. Demultiplexed fastq file was split into sample-specific fastq files using split_sequence_file_on_sample_ids.py script from QIIME2. Individual fastq files without non-biological nucleotides were processed using Divisive Amplicon Denoising Algorithm (DADA) pipeline. The output of the dada2 pipeline (feature table of amplicon sequence variants (an ASV table)) was processed for alpha and beta diversity analysis using *phyloseq*, and microbiomeSeq (http://www.github.com/umerijaz/microbiomeSeq) packages in R. We analyzed variance (ANOVA) among sample categories while measuring the of α-diversity measures using plot_anova_diversity function in *microbiomeSeq* package. Permutational multivariate analysis of variance (PERMANOVA) with 999 permutations was performed on all principal coordinates obtained during CCA with the *ordination* function of the *microbiomeSeq* package. Pairwise correlation was performed between the microbiome (genera) and metabolomics (metabolites) data was performed using the microbiomeSeq package.

### Quantification and statistical analysis

The Wilcoxon signed rank test was used to determine the statistical differences between α diversity patterns. White’s non-parametric t-test and analysis of variance (ANOVA) post hoc analysis were performed. A Semi-Parametric Rank-based approach for Inference in a Graphic model (SPRING) from NetCoMi (Network Construction and comparison for Microbiome data) package in R was used to perform network analysis. Node sizes were scaled using Eigenvector centrality. Differential abundance test benchmarking was performed using DAtest package (https://github.com/Russel88/DAtest/wiki/usage#typical-workflow). Briefly, differentially abundant methods were compared with False Discovery Rate (FDR), Area Under the (Receiver Operator) Curve (AUC), Empirical power (Power), and False Positive Rate (FPR). Based on the DAtest’s benchmarking, we selected lefseq and ANOVA as the methods of choice to perform differential abundance analysis. We assessed the statistical significance (*p* < 0.05) throughout. Whenever necessary, we adjusted *p*-values for multiple comparisons according to the Benjamini and Hochberg method to control False Discovery Rate (Benjamini and Hochberg, 1995).

SAS/STAT version 9.4 PROC POWER was used to perform power calculations.

## References

[1] (2021). WHO. https://www.who.int/news-room/fact-sheets/detail/levels-and-trends-in-child-mortality-report-2021.

[2] Ahmed B., Abushama M., Konje J.C. (2023). Prevention of spontaneous preterm delivery - an update on where we are today. J. Matern. Fetal Neonatal Med..

[3] Walani S.R. (2020). Global burden of preterm birth. Int. J. Gynaecol. Obstet..

[4] Ananth C.V., Goldenberg R.L., Friedman A.M., Vintzileos A.M. (2018). Association of temporal changes in gestational age with perinatal mortality in the United States, 2007-2015. JAMA Pediatr..

[5] Ansari A., You Y.A., Lee G., Kim S.M., Park S.W., Hur Y.M., Kim Y.J. (2024). Dysbiotic vaginal microbiota induces preterm birth cascade via pathogenic molecules in the vagina. Metabolites.

[6] Gorczyca K., Obuchowska A., Kimber-Trojnar Ż., Wierzchowska-Opoka M., Leszczyńska-Gorzelak B. (2022). Changes in the gut microbiome and pathologies in pregnancy. Int. J. Environ. Res. Public Health.

[7] Ursell L.K., Metcalf J.L., Parfrey L.W., Knight R. (2012). Defining the human microbiome. Nutr. Rev..

[8] Turnbaugh P.J., Ley R.E., Hamady M., Fraser-Liggett C.M., Knight R., Gordon J.I. (2007). The human microbiome project. Nature.

[9] Yao Y., Cai X., Ye Y., Wang F., Chen F., Zheng C. (2021). The role of microbiota in infant health: From early life to adulthood. Front. Immunol..

[10] Tadros J.S., Llerena A., Sarkar A., Johnson R., Miller E.M., Gray H.L., Ho T.T.B. (2022). Postnatal growth and gut microbiota development influenced early childhood growth in preterm infants. Front. Pediatr..

[11] Fung T.C., Olson C.A., Hsiao E.Y. (2017). Interactions between the microbiota, immune and nervous systems in health and disease. Nat. Neurosci..

[12] Houghteling P.D., Walker W.A. (2015). Why is initial bacterial colonization of the intestine important to infants’ and children’s health?. J. Pediatr. Gastroenterol. Nutr..

[13] Niu J., Xu L., Qian Y., Sun Z., Yu D., Huang J., Zhou X., Wang Y., Zhang T., Ren R. (2020). Evolution of the gut microbiome in early childhood: a cross-sectional study of Chinese children. Front. Microbiol..

[14] Chen Y., Lu Y., Wang T., Wu J., Yu B. (2023). Changes in gut microbiota at 1-60 days in 92 preterm infants in a neonatal intensive care unit using 16S rRNA gene sequencing. Med. Sci. Monit..

[15] Dalby M.J., Hall L.J. (2020). Recent advances in understanding the neonatal microbiome. F1000Res..

[16] Asadi S., Bloomfield F.H., Harding J.E. (2019). Nutrition in late preterm infants. Semin. Perinatol..

[17] Wilcox A.J. (2001). On the importance--and the unimportance--of birthweight. Int. J. Epidemiol..

[18] Harris S.R. (2015). Measuring head circumference: Update on infant microcephaly. Can. Fam. Physician.

[19] Gough E.K., Edens T.J., Geum H.M., Baharmand I., Gill S.K., Robertson R.C., Mutasa K., Ntozini R., Smith L.E., Chasekwa B. (2021). Maternal fecal microbiome predicts gestational age, birth weight and neonatal growth in rural Zimbabwe. EBioMedicine.

[20] AAP (2022). Preterm infant growth tools. https://www.aap.org/en/patient-care/newborn-and-infant-nutrition/newborn-and-infant-nutrition-assessment-tools/preterm-infant-growth-tools/.

[21] Benson T.W., Conrad K.A., Li X.S., Wang Z., Helsley R.N., Schugar R.C., Coughlin T.M., Wadding-Lee C., Fleifil S., Russell H.M. (2023). Gut microbiota-derived Trimethylamine N-Oxide contributes to abdominal aortic aneurysm through inflammatory and apoptotic mechanisms. Circulation.

[22] Chambers L.M., Esakov Rhoades E.L., Bharti R., Braley C., Tewari S., Trestan L., Alali Z., Bayik D., Lathia J.D., Sangwan N. (2022). Disruption of the gut microbiota confers cisplatin resistance in epithelial ovarian cancer. Cancer Res..

[23] Hamidi M., Cruz-Lebrón A., Sangwan N., Blatz M.A., Levine A.D. (2023). Maternal vertical microbial transmission during skin-to-skin care. Adv. Neonatal Care.

[24] Lundy S.D., Sangwan N., Parekh N.V., Selvam M.K.P., Gupta S., McCaffrey P., Bessoff K., Vala A., Agarwal A., Sabanegh E.S. (2021). Functional and taxonomic dysbiosis of the gut, urine, and semen microbiomes in male infertility. Eur. Urol..

[25] Osborn L.J., Orabi D., Goudzari M., Sangwan N., Banerjee R., Brown A.L., Kadam A., Gromovsky A.D., Linga P., Cresci G.A.M. (2021). A Single human-relevant fast food meal rapidly reorganizes metabolomic and transcriptomic signatures in a gut microbiota-dependent manner. Immunometabolism.

[26] Traughber C.A., Iacano A.J., Neupane K., Khan M.R., Opoku E., Nunn T., Prince A., Sangwan N., Hazen S.L., Smith J.D., Gulshan K. (2023). Impavido attenuates inflammation, reduces atherosclerosis, and alters gut microbiota in hyperlipidemic mice. iScience.

[27] Tzeng A., Sangwan N., Jia M., Liu C.C., Keslar K.S., Downs-Kelly E., Fairchild R.L., Al-Hilli Z., Grobmyer S.R., Eng C. (2021). Human breast microbiome correlates with prognostic features and immunological signatures in breast cancer. Genome Med..

[28] Zhang S., Han Y., Schofield W., Nicosia M., Karell P.E., Newhall K.P., Zhou J.Y., Musich R.J., Pan S., Valujskikh A. (2023). Select symbionts drive high IgA levels in the mouse intestine. Cell Host Microbe.

[29] Zhu Y., Dwidar M., Nemet I., Buffa J.A., Sangwan N., Li X.S., Anderson J.T., Romano K.A., Fu X., Funabashi M. (2023). Two distinct gut microbial pathways contribute to meta-organismal production of phenylacetylglutamine with links to cardiovascular disease. Cell Host Microbe.

[30] DADA2 pipeline 1.8 [Internet]. Available from: https://benjjneb.github.io/dada2/tutorial_1_8.html.

[31] Zhang Y., Parmigiani G., Johnson W.E. (2020). ComBat-seq: Batch effect adjustment for RNA-seq count data. NAR Genom. Bioinform..

[32] Peschel S., Müller C.L., von Mutius E., Boulesteix A.L., Depner M. (2021). NetCoMi: network construction and comparison for microbiome data in R. Brief. Bioinform..

[33] Baud A., Hillion K.H., Plainvert C., Tessier V., Tazi A., Mandelbrot L., Poyart C., Kennedy S.P. (2023). Microbial diversity in the vaginal microbiota and its link to pregnancy outcomes. Sci. Rep..

[34] Shin H., Martinez K.A., Henderson N., Jay M., Schweizer W., Bogaert D., Park G., Bokulich N.A., Blaser M.J., Dominguez-Bello M.G. (2023). Partial convergence of the human vaginal and rectal maternal microbiota in late gestation and early post-partum. NPJ Biofilms Microbiomes.

[35] Park S., You Y.A., Kim Y.H., Kwon E., Ansari A., Kim S.M., Lee G., Hur Y.M., Jung Y.J., Kim K., Kim Y.J. (2022). Ureaplasma and Prevotella colonization with Lactobacillus abundance during pregnancy facilitates term birth. Sci. Rep..

[36] Alinezhad S., Bakhshandehnosrat S., Baniaghil A.S., Livani S., Bazouri M., Shafipour M., Behnampour N., Ghaemi E.A. (2022). The role of genital Mycoplasmas in preterm labor. J. Reprod. Infertil..

[37] Mao L., Gao B., Chang H., Shen H. (2024). Interaction and Metabolic Pathways: Elucidating the role of gut microbiota in gestational diabetes mellitus pathogenesis. Metabolites.

[38] Dos Anjos Borges L.G., Pastuschek J., Heimann Y., Dawczynski K., Schleußner E., Pieper D.H., Zöllkau J., PEONS study group (2023). Vaginal and neonatal microbiota in pregnant women with preterm premature rupture of membranes and consecutive early onset neonatal sepsis. BMC Med..

[39] Wang J., Zheng J., Shi W., Du N., Xu X., Zhang Y., Ji P., Zhang F., Jia Z., Wang Y. (2018). Dysbiosis of maternal and neonatal microbiota associated with gestational diabetes mellitus. Gut.

[40] Baldassarre M.E., Di Mauro A., Capozza M., Rizzo V., Schettini F., Panza R., Laforgia N. (2019). Dysbiosis and prematurity: Is there a role for probiotics?. Nutrients.

[41] Group B Strep (GBS) [Internet]. Available from: https://www.cdc.gov/group-b-strep/symptoms/index.html

[42] Mei J.Y., Silverman N.S. (2023). Group B Streptococcus in pregnancy. Obstet. Gynecol. Clin. North Am..

[43] Menichini D., Chiossi G., Monari F., De Seta F., Facchinetti F. (2022). Supplementation of probiotics in pregnant women targeting group B Streptococcus colonization: A systematic review and meta-analysis. Nutrients.

[44] Dugoua J.J., Machado M., Zhu X., Chen X., Koren G., Einarson T.R. (2009). Probiotic safety in pregnancy: a systematic review and meta-analysis of randomized controlled trials of Lactobacillus, Bifidobacterium, and Saccharomyces spp. J. Obstet. Gynaecol. Can..

[45] Hayes K., Janssen P., Payne B.A., Jevitt C., Johnston W., Johnson P., Butler M. (2024). Oral probiotic supplementation in pregnancy to reduce group B Streptococcus colonisation (OPSiP trial): Study protocol for a double-blind parallel group randomised placebo trial. BMJ Open.

[46] Arboleya S., Solís G., Fernández N., de los Reyes-Gavilán C.G., Gueimonde M. (2012). Facultative to strict anaerobes ratio in the preterm infant microbiota: A target for intervention?. Gut Microb..

[47] Terrazzan Nutricionist A.C., Procianoy R.S., Roesch L.F.W., Corso A.L., Dobbler P.T., Silveira R.C. (2020). Meconium microbiome and its relation to neonatal growth and head circumference catch-up in preterm infants. PLoS One.

[48] Shin H., Pei Z., Martinez K.A., Rivera-Vinas J.I., Mendez K., Cavallin H., Dominguez-Bello M.G. (2015). The first microbial environment of infants born by C-section: The operating room microbes. Microbiome.

[49] Cassidy-Bushrow A.E., Sitarik A., Levin A.M., Lynch S.V., Havstad S., Ownby D.R., Johnson C.C., Wegienka G. (2016). Maternal group B Streptococcus and the infant gut microbiota. J. Dev. Orig. Health Dis..

[50] Dominguez-Bello M.G., De Jesus-Laboy K.M., Shen N., Cox L.M., Amir A., Gonzalez A., Bokulich N.A., Song S.J., Hoashi M., Rivera-Vinas J.I. (2016). Partial restoration of the microbiota of cesarean-born infants via vaginal microbial transfer. Nat. Med..

[51] Zhang C., Li L., Jin B., Xu X., Zuo X., Li Y., Li Z. (2021). The effects of delivery mode on the gut microbiota and health: State of art. Front. Microbiol..

[52] Zhou L., Qiu W., Wang J., Zhao A., Zhou C., Sun T., Xiong Z., Cao P., Shen W., Chen J. (2023). Effects of vaginal microbiota transfer on the neurodevelopment and microbiome of cesarean-born infants: A blinded randomized controlled trial. Cell Host Microbe.

[53] Gregory K.E., LaPlante R.D., Shan G., Kumar D.V., Gregas M. (2015). Mode of birth influences preterm infant intestinal colonization with Bacteroides over the early neonatal period. Adv. Neonatal Care.

[54] Amir M., Brown J.A., Rager S.L., Sanidad K.Z., Ananthanarayanan A., Zeng M.Y. (2020). Maternal microbiome and infections in pregnancy. Microorganisms.

[55] You Y.A., Yoo J.Y., Kwon E.J., Kim Y.J. (2019). Blood microbial communities during pregnancy are associated with preterm birth. Front. Microbiol..

[56] Collado M.C., Isolauri E., Laitinen K., Salminen S. (2008). Distinct composition of gut microbiota during pregnancy in overweight and normal-weight women. Am. J. Clin. Nutr..

[57] Dreisbach C., Prescott S., Siega-Riz A.M., McCulloch J., Habermeyer L., Dudley D., Trinchieri G., Kelsey C., Alhusen J. (2023). Composition of the maternal gastrointestinal microbiome as a predictor of neonatal birth weight. Pediatr. Res..

[58] Sonagra A.D., Biradar S.M., K D., Murthy D S J. (2014). Normal pregnancy- a state of insulin resistance. J. Clin. Diagn. Res..

[59] Andreani G.A., Mahmood S., Patel M.S., Rideout T.C. (2023). Maternal pea fiber supplementation to a high calorie diet in obese pregnancies protects male offspring from metabolic dysfunction in adulthood. J. Dev. Orig. Health Dis..

[60] Kuang J., Sun S., Ke F. (2023). The effects of exercise intervention on complications and pregnancy outcomes in pregnant women with overweight or obesity: A systematic review and meta-analysis. Medicine (Baltim.).

[61] Monda V., Villano I., Messina A., Valenzano A., Esposito T., Moscatelli F., Viggiano A., Cibelli G., Chieffi S., Monda M., Messina G. (2017). Exercise modifies the gut microbiota with positive health effects. Oxid. Med. Cell. Longev..

[62] Martín-Grau C., Díaz-López A., Aparicio E., Arija V. (2022). Short-chain fatty acid reference ranges in pregnant women from a Mediterranean region of Northern Spain: ECLIPSES Study. Nutrients.

[63] den Besten G., van Eunen K., Groen A.K., Venema K., Reijngoud D.J., Bakker B.M. (2013). The role of short-chain fatty acids in the interplay between diet, gut microbiota, and host energy metabolism. J. Lipid Res..

[64] Joubert I.A., Otto M., Strunk T., Currie A.J. (2022). Look who’s talking: Host and pathogen drivers of Staphylococcus epidermidis virulence in neonatal sepsis. Int. J. Mol. Sci..

[65] Dong Y., Glaser K., Speer C.P. (2018). New Threats from an old foe: Methicillin-resistant Staphylococcus aureus Infections in neonates. Neonatology.

[66] Yu Y., Lu L., Sun J., Petrof E.O., Claud E.C. (2016). Preterm infant gut microbiota affects intestinal epithelial development in a humanized microbiome gnotobiotic mouse model. Am. J. Physiol. Gastrointest. Liver Physiol..

[67] Jena A., Montoya C.A., Mullaney J.A., Dilger R.N., Young W., McNabb W.C., Roy N.C. (2020). Gut-brain axis in the early postnatal years of life: A developmental perspective. Front. Integr. Neurosci..

[68] Ortiz-Huidobro R.I., Larqué C., Velasco M., Chávez-Maldonado J.P., Sabido J., Sanchez-Zamora Y.I., Hiriart M. (2022). Sexual dimorphism in the molecular mechanisms of insulin resistance during a critical developmental window in Wistar rats. Cell Commun. Signal..

[69] Hartz L.E., Bradshaw W., Brandon D.H. (2015). Potential NICU environmental influences on the neonate's microbiome: A systematic review. Adv. Neonatal Care.

