# Supplementary material for: Maternal and infant microbiome and birth anthropometry
Source: iScience. 2024 Jun 21;27(10):110312. doi: 10.1016/j.isci.2024.110312 (PMC11462025; doi:10.1016/j.isci.2024.110312)
Supplement: Document S1. Figures S1 and S2 [file mmc1.pdf]

iScience, Volume *27*

Supplemental information

Maternal and infant microbiome and birth anthropometry

Swetha Padiyar, Vanishree Nandakumar, Swapna Kollikonda, Sreenivas Karnati, Naseer Sangwan, and Hany Aly

Supplemental figure legends

Supplemental figure 1A: Maternal microbial differences in gestational morbidities.

Relative abundancies in patients with hypertension (A) are indicated in orange (gestational hypertension) Abundancies in preterm premature rupture of membranes (B) and preeclampsia (C) are indicated in green and orange indicates abundancies in relatively healthy mothers.

Supplemental figure 1 B: Maternal microbial differences in gestational morbidities

Relative abundancies in patients with Diabetes (D) are indicated in green (Type 1) and purple (gestational diabetes). Abundancies in Infections (E) and Chorioamnionitis (F) are indicated in green; orange indicates abundancies in relatively healthy mothers.

Supplemental Figure 2: Differential abundance of infant's microbiota based on mode of delivery

Blue indicates abundancies in infants born via cesarian section (C-section) and red indicates abundancies in infants born vaginally

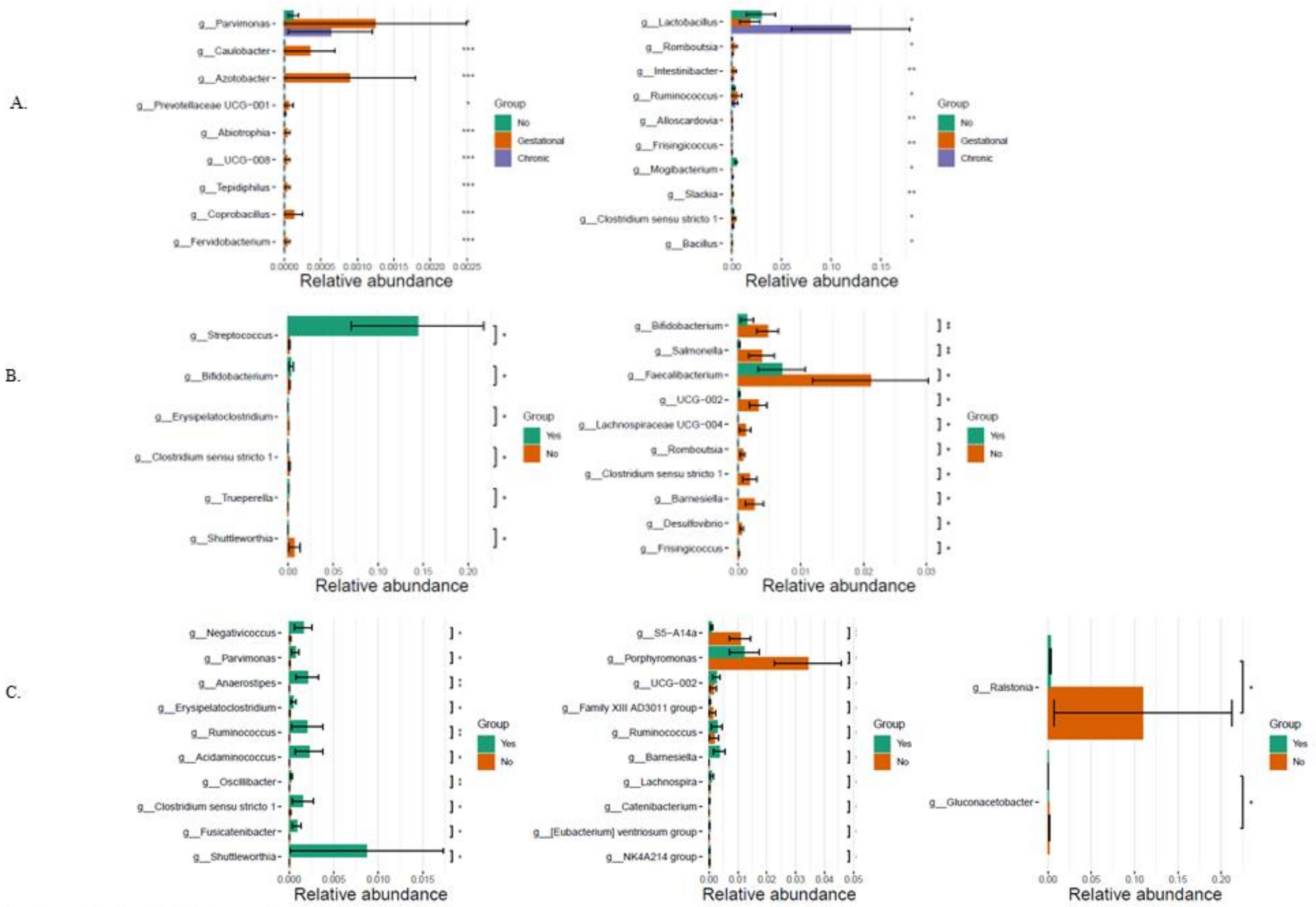

Supplemental figure 1A: Maternal microbial differences in gestational morbidities.

Relative abundancies in patients with hypertension (A) are indicated in orange (gestational hypertension) Abundancies in preterm premature rupture of membranes (B) and preeclampsia (C) are indicated in green, and orange indicates abundancies in relatively healthy mothers.

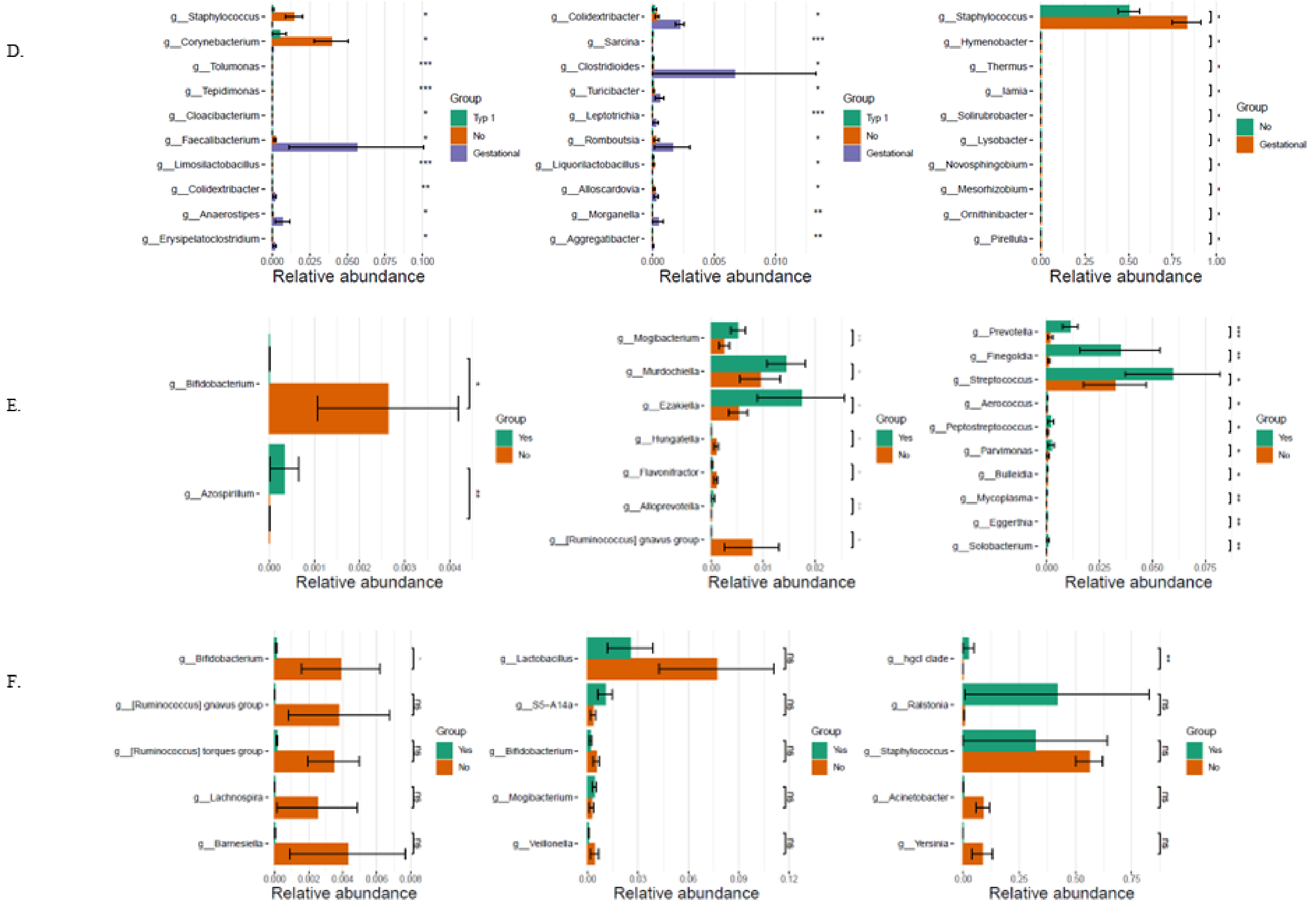

Supplemental figure 1 B: Maternal microbial differences in gestational morbidities

Relative abundancies in patients with Diabetes (D) are indicated in green (Type 1) and purple (gestational diabetes). Abundancies in Infections (E) and Chorioamnionitis (F) are indicated in green; orange indicates abundancies in relatively healthy mothers.

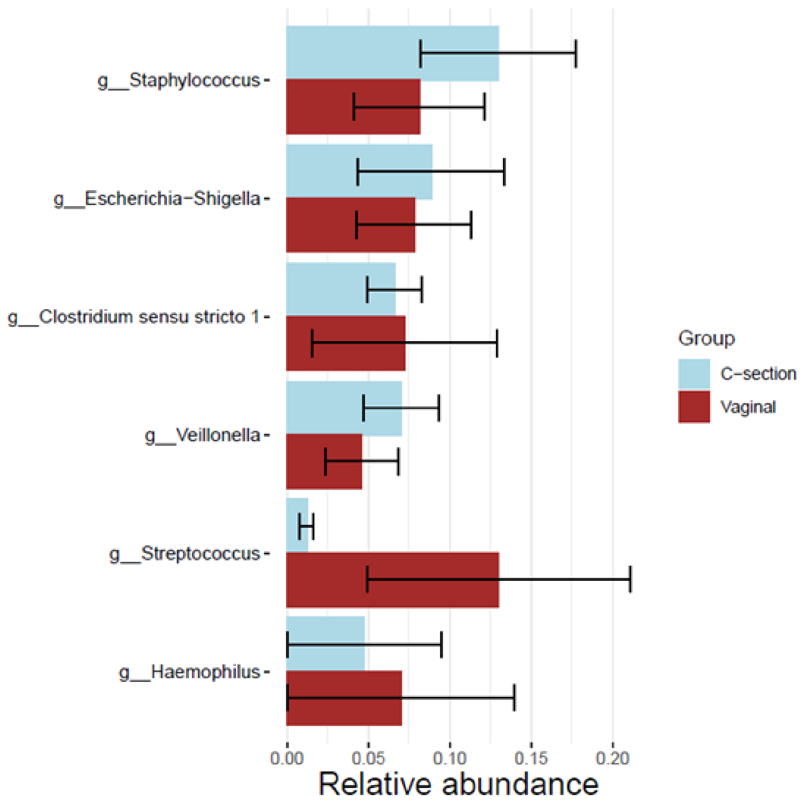

Supplemental Figure 2: Differential abundance of infant's microbiota based on mode of delivery
Blue indicates abundancies in infants born via cesarian section (C-section) and red indicates abundancies in infants born vaginally
